# Editorial: Education in pharmacology of anti-cancer drugs: 2023

**DOI:** 10.3389/fphar.2024.1422847

**Published:** 2024-05-20

**Authors:** João G. Costa, Olivier Feron, Ana Sofia Fernandes

**Affiliations:** ^1^ CBIOS, Universidade Lusófona Research Center for Biosciences & Health Technologies, Lisbon, Portugal; ^2^ Pole of Pharmacology and Therapeutics (FATH), Institut de Recherche Expérimentale et Clinique (IREC), UCLouvain, Brussels, Belgium; ^3^ Walloon Excellence in Life Sciences and Biotechnology (WELBIO) Department, WEL Research Institute, Wavre, Belgium

**Keywords:** education, cancer, cancer models, bibliometric research, network pharmacolgy, biosimilar

In the fight against cancer, education stands as a powerful ally. Understanding the pharmacology of anti-cancer drugs has profound implications for patient-centric care. In recent decades, the landscape of cancer treatment has witnessed remarkable advancements. The identification of novel therapeutic targets and treatment strategies, such as monoclonal antibodies, immune checkpoint inhibitors, cell therapies, or anti-tumor vaccines, has revolutionized cancer pharmacology. Education in pharmacology is therefore essential to equip researchers and healthcare professionals with the knowledge necessary to drive groundbreaking discoveries and optimize therapeutic outcomes. By understanding drug metabolism, distribution, and elimination, clinicians can tailor dosing regimens to individual patients. Moreover, insights into pharmacodynamics enable precise targeting of cancer cells while sparing healthy tissue, a critical aspect of mitigating side effects. Furthermore, through education, healthcare providers can anticipate resistance patterns, implement appropriate strategies to circumvent or overcome them, and proactively adjust treatment plans, thereby prolonging patient survival and improving quality of life. Beyond healthcare professionals, patient education is equally essential. Informed patients become active participants in their treatment journey, empowered to make educated decisions and adhere to prescribed regimens.

As far as research is concerned, education can also be a catalyst for innovation. By fostering a culture of continuous learning and inquiry, education propels the field of oncology forward. In particular, new tools derived from systems biology approaches are currently paving the way for the attractiveness of network pharmacology for educational purposes. Network pharmacology is an interdisciplinary field that integrates principles from pharmacology and computational biology to understand the complex interactions between drugs, targets, and biological systems at the network level. Network pharmacology uses analytical techniques to represent the interactions between drugs, targets, genes, proteins, metabolites, and other biological entities. By constructing and analyzing these networks, researchers can identify key nodes (e.g., drug targets, signaling pathways) and understand the overall structure and dynamics of the system. In this Research Topic (RT), Han et al. (2023) described a bibliometric analysis that examines hotspots and trends in the field of network pharmacology research in cancer treatment. The authors addressed the challenges of network pharmacology, including the need for advanced computational and statistical methods and the limited availability of comprehensive and accurate drug-target interaction databases. They also emphasized the absolute need for cross-validation to improve the accuracy and reliability of holistic approaches by filtering out low-quality or inconsistent data. In a second study, Han et al. (2024) applied bibliometric analysis to comprehensively characterize the current state of research on drug resistance in head and neck cancer, identifying trends and pointing out research directions. This approach is still in its infancy, and some biases in such early studies are inevitable. However, the ongoing refinement of methodological approaches by bioinformaticians specifically trained in the field of medical oncology will undoubtedly offer substantial support to anti-cancer drug research.

Network pharmacology also provides tools to enable the repurposing of existing drugs for novel therapeutic indications, in addition to insights into drug biosimilarity. A biosimilar drug is a biological product that is highly similar to a previously approved biological medicine in terms of quality, safety, and efficacy. Unlike generic drugs, which are exact copies of their chemical counterparts (small-molecule drugs), biosimilars are not identical to the reference product due to the inherent complexity of biological molecules and the variability that can arise during manufacturing. Park et al. (2023) evaluated the efficacy of SB8, a biosimilar to the reference biologic bevacizumab, through simulations based on a time-to-event (TTE) model for overall survival (OS) and progression-free survival (PFS), in non-small cell lung cancer (NSCLC) using data from a comparative clinical trial, a phase III study. The use of SB8 did not significantly affect OS and PFS hazards, showing similar simulated values. Overall, the study demonstrated comparable efficacy between SB8 and bevacizumab in advanced NSCLC based on their exposure levels.

In a broader sense, network pharmacology acknowledges the multi-target nature of drugs, unlike traditional drug development methods that prioritize single drug-single target approaches. This aspect is often overlooked or underemphasized by drug developers, partly for the sake of simplifying communication. Polypharmacology refers to the phenomenon whereby a drug interacts with multiple targets or pathways within biological systems, potentially leading to enhanced therapeutic efficacy and broader clinical applications. Polypharmacology becomes particularly intriguing when considering tumor heterogeneity, which can lead to different responses to treatments within a single tumor. Indeed, this underscores the critical need for appropriate *in vivo* models for drug testing. In this RT, Li et al. (2024) delved into the rationale behind current preclinical tumor models used to assess drug efficacy, suggesting that targeting tumor recurrence and progression, rather than just tumor growth, is crucial to enhancing the prognosis of cancer patients with anti-tumor treatments. Additionally, these authors explored the limitations of conventional preclinical tumor models in evaluating efficacy and proposed the orthotopic tumor transplantation and resection model as a novel approach to mimic tumor recurrence and metastasis. The strategies outlined in this study, by improving the accuracy of efficacy evaluation, offer fresh perspectives for refining and accelerating the development of anti-cancer drugs or therapies, optimizing treatment protocols to improve OS, and reducing drug development and cancer treatment costs.

In conclusion, this RT highlights the importance of utilizing technology-driven analytical methods while also refining experimental models to monitor the effectiveness of anti-cancer drugs. It is crucial to provide researchers with this dual understanding, encompassing both computational and experimental knowledge, so as to drive innovation and improve patient outcomes. Investments in education, including curriculum enhancements and lifelong learning for health professionals, are vital to accomplishing these objectives.

